# Syncope in an Adolescent

**DOI:** 10.7759/cureus.6669

**Published:** 2020-01-15

**Authors:** Hardik Patel, Nick Chatla, Ahmad Khiami

**Affiliations:** 1 Pediatrics, Raleigh General Hospital, Beckley, USA

**Keywords:** syncope, mycoplasma pneumoniae, community acquired pneumonia

## Abstract

Syncope is a common chief complaint in the emergency department (ED); however, the workup of syncope is quite extensive. Likewise, community-acquired pneumonia (CAP) can also be difficult to diagnose due to the wide range of signs and symptoms, and even a larger list of etiologies. Syncope as a presenting symptom of CAP has demonstrated to be a challenging clinical association to make. We present a case of a previously healthy 14-year-old female who presented to the ED for having an episode of syncope at softball practice. Notable aspects of the case-age of the patient and the obscure presentation, as well as laboratory and imaging results-made this case challenging. Thus, obtaining a thorough history from the patient and family and performing a detailed physical examination can really help a clinician lead to an accurate diagnosis.

## Introduction

Syncope is a common chief complaint in the emergency department (ED); however, the workup of syncope is quite extensive. Defined as a brief loss of consciousness caused by hypoperfusion of the brain, syncope has many causes, including cardiogenic, vasovagal, orthostatic, and neurologic, and is associated with a vast differential diagnosis. Yet, up to a third of syncopal episodes have an unknown etiology. We present a case of a healthy adolescent who presented to the ED after having an episode of syncope and will review the diagnostic considerations and pathophysiology behind the final diagnosis.

## Case presentation

A healthy 14-year-old female was brought to the ED after experiencing a syncopal episode at softball practice. She did not recall experiencing any dizziness, chest pain, shortness of breath, palpitations, or trauma prior to the event. Within approximately one minute, she became nauseated and started experiencing chest pressure, shortness of breath, non-productive cough, and trace amount of blood coming out of her mouth. She was aware of having a syncopal episode and denied being confused. Per bystanders, the patient’s eyes rolled into the back of her head, but she did not have seizure activity. She endorsed starting birth control pills two months prior. Last menstrual period was one week ago, regular, lasted three to four days, and without heavy flow. The patient mentioned that she skipped lunch before practice; however, she denies any recent dieting, allergies, over-the-counter or illicit drug use, past medical problems, or surgical history.

Vital signs upon presentation were as follows: temperature of 98.0o F, pulse rate of 123 beats per minute, respiratory rate of 18 breaths per minute, blood pressure of 130/78 mmHg, and oxygen saturation of 98% on room air. Physical examination demonstrates the following:

● General: awake, alert and oriented, well-nourished, and in no acute distress.

● HEENT (head, eyes, ears, nose, and throat): the head is normocephalic and atraumatic. Pupils are equal, round, and reactive to light and accommodation; extraocular muscles are intact. Ear, nose, and throat present with moist, mucous membrane with no active bleeding.

● Cardiovascular: tachycardia with regular rhythm but without murmurs.

● Pulmonary: clear breath sounds, except for trace wheezing in the right middle lobe.

● Abdomen: Soft, non-tender, non-distended, and without organomegaly.

● Musculoskeletal: grossly normal; full range of motion without edema in the upper or lower extremities.

**Figure 1:**
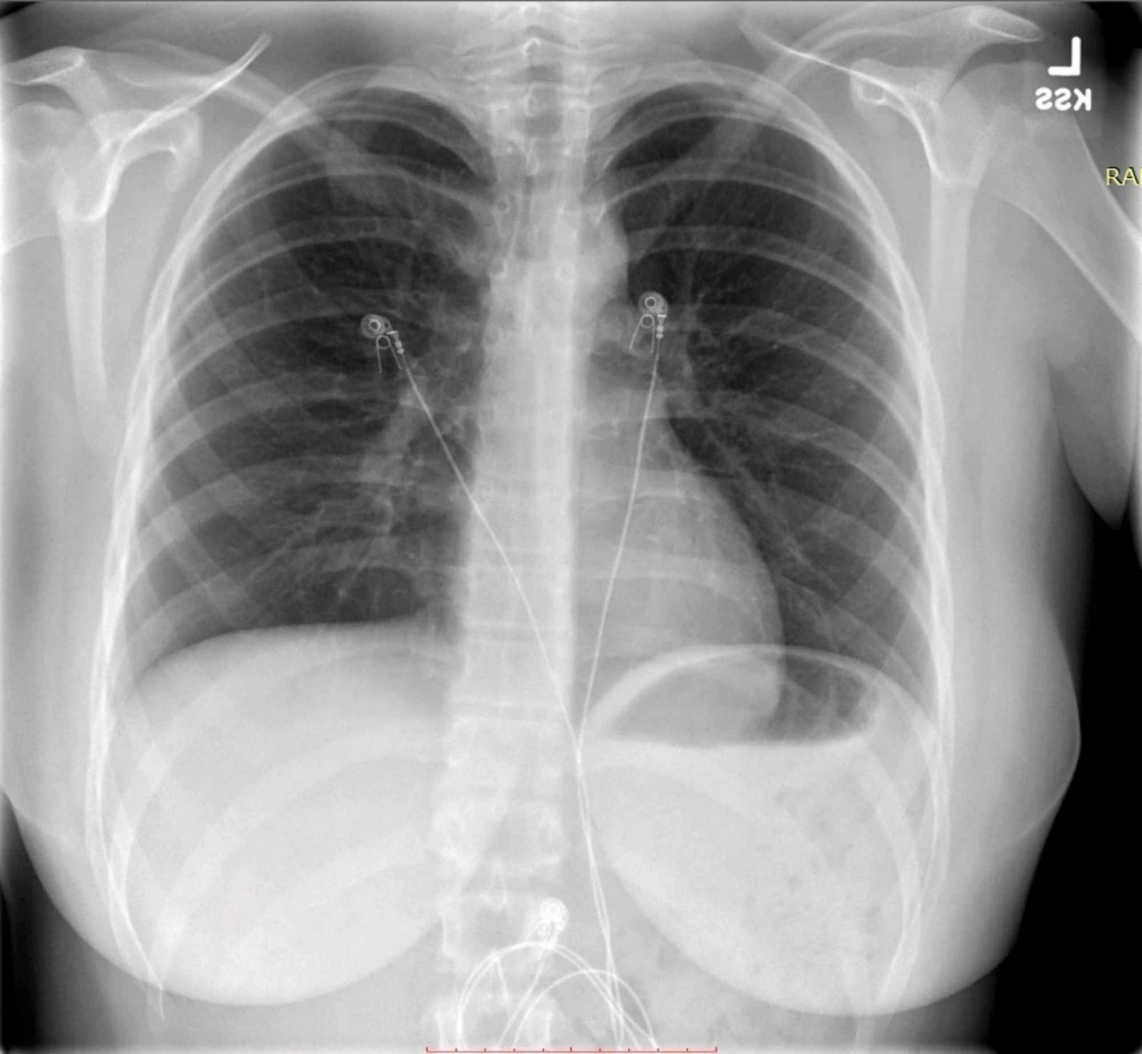
Two-view chest X-ray (anteroposterior view) showing no evidence of acute cardiopulmonary process

**Figure 2:**
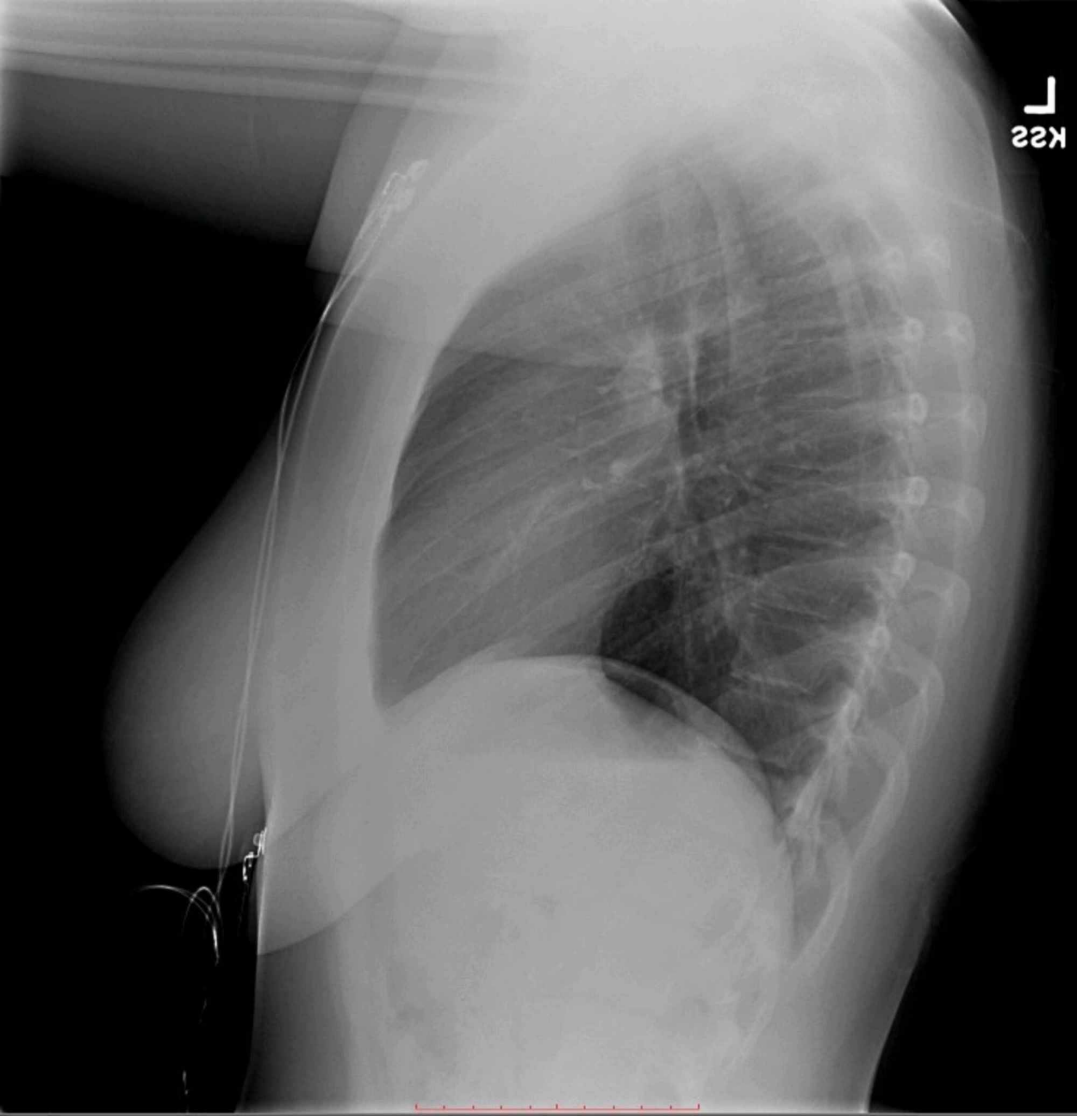
Two-view chest X-ray (lateral view) showing no evidence of acute cardiopulmonary process

**Figure 3:**
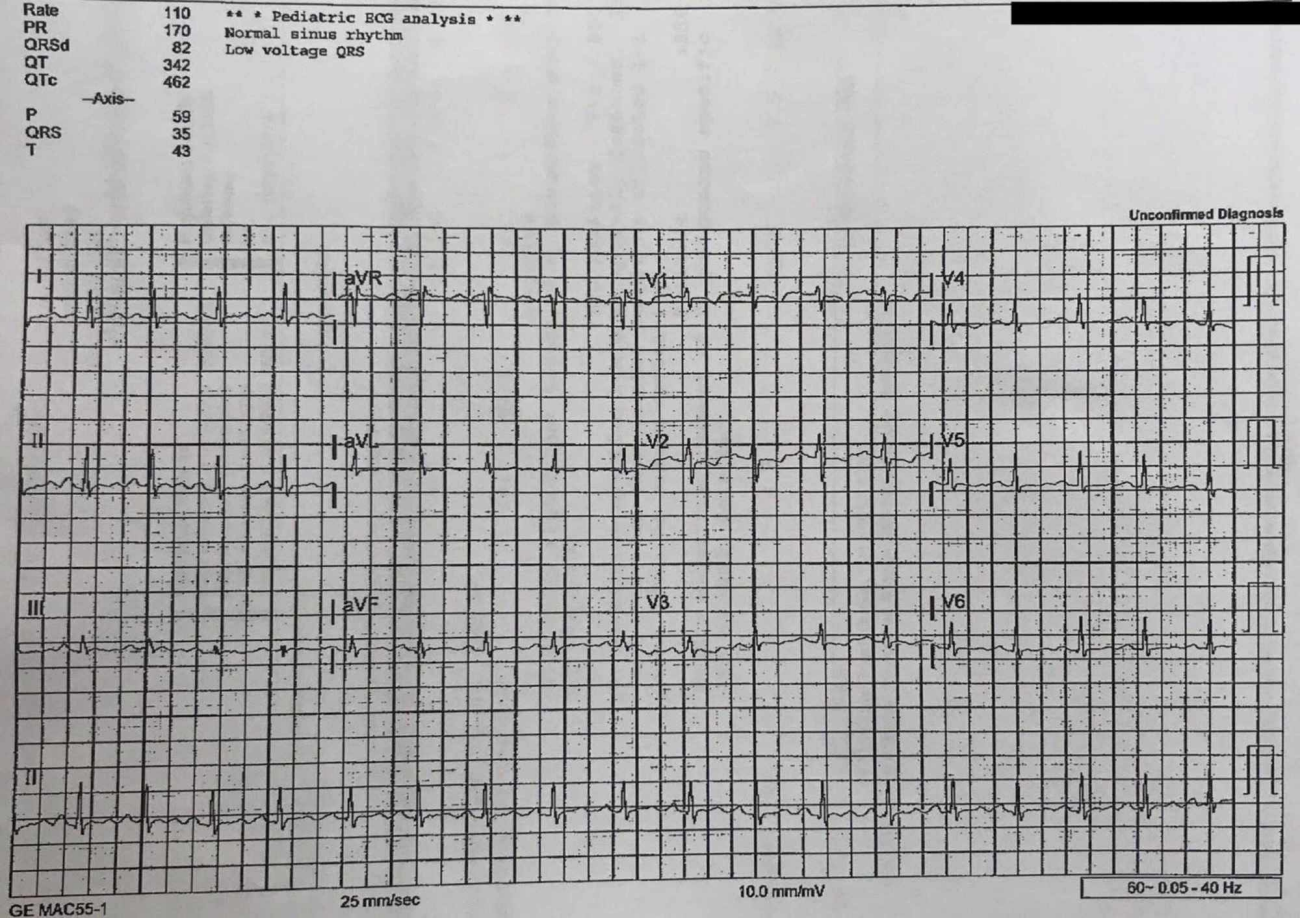
Electrocardiogram at the emergency department showing sinus tachycardia

**Figure 4:**
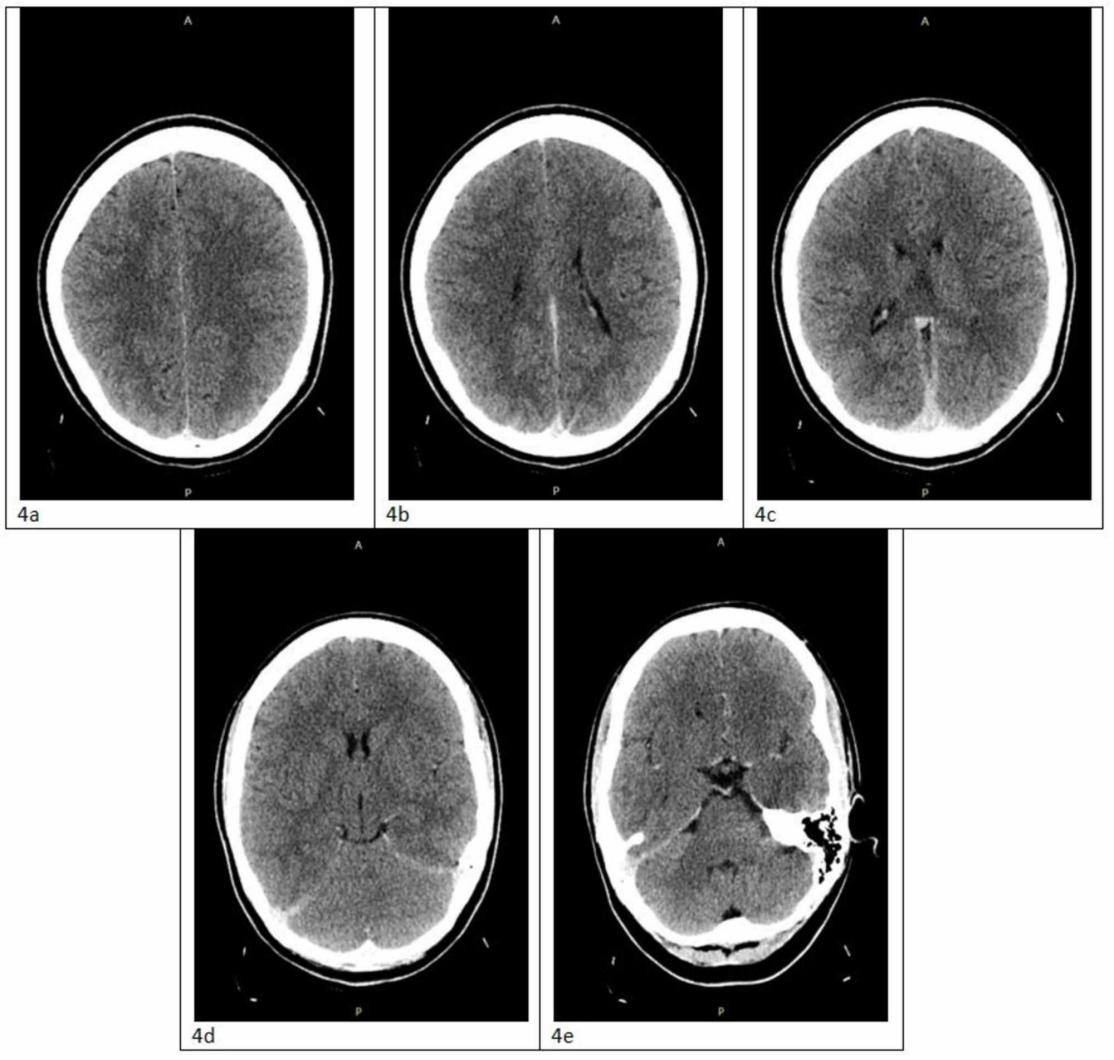
CT of the brain without contrast within normal limits (a-e) Views of the brain in different transverse planes (top to bottom)

**Figure 5:**
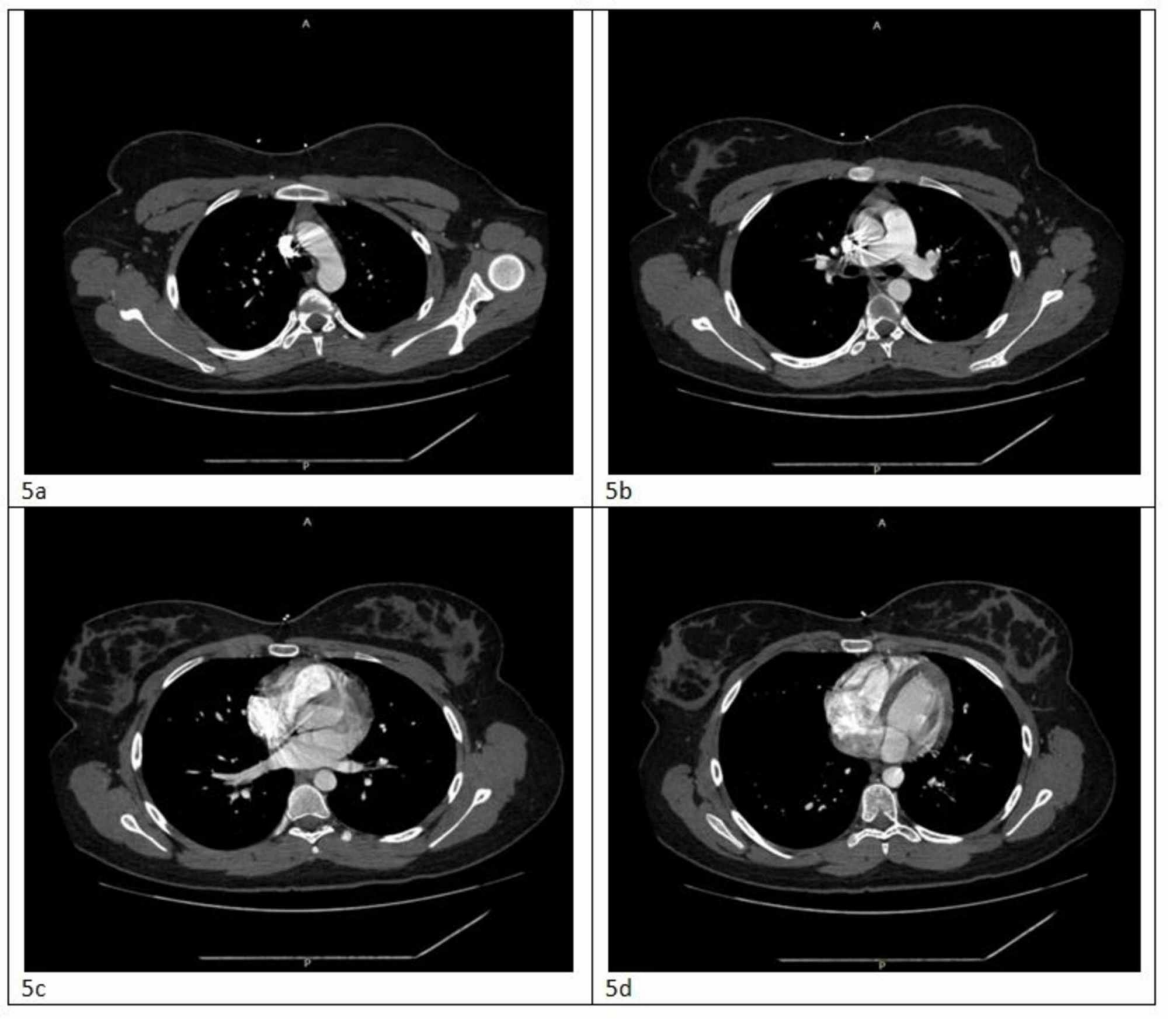
CT angiography of chest PE within normal limits (a-d) Views of the chest in different transverse planes (top to bottom) PE, pulmonary emboli

Laboratory studies at the ED showed a D-dimer count of 3.24 mg/L (normal range: 0.19-0.59 mg/L). All other studies including, troponin, complete blood count, complete metabolic panel, and HCG (human chorionic gonadotropin) serum quantity were within normal limits. Subsequent tests of brain natriuretic peptide, lactic acid, procalcitonin, urine analysis, and urine drug screen were also within normal limits (Table 1-5). Two-view chest X-ray was normal, and EKG (electrocardiogram) showed sinus tachycardia (Figures 1-3). CT of the brain without contrast was normal, and CT angiography of the chest did not show pulmonary emboli (Figures 4, 5).

 

**Table 1:** Complete metabolic panel showing normal results

Test	Result	Reference	Units
Sodium (Na)	139	137-144	mmol/L
Potassium (K)	3.8	3.5-5.0	mmol/L
Chloride (Cl)	108	98-108	mmol/L
Carbon dioxide (CO_2_)	25	21-32	mmol/L
Anion gap	10	0-40	mmol/L
Glucose	101	55-115	mg/dL
Blood urea nitrogen (BUN)	12	7-18	mg/dL
Creatinine	0.9	0.6-1.3	mg/dL
Total protein	7.7	6.4-8.2	g/dL
Albumin	4.2	3.4-5.0	G/dL
Globulin	3.5 (H)	2.1-3.4	g/dL
Albumin/globulin ratio	1.2	1.0-2.6	
Calcium	9.8	8.5-10.1	mg/dL
Total bilirubin	0.4	0.0-5.0	mg/dL
Aspartate aminotransferase (SGOT/AST)	15	15-37	units/L
Alanine aminotransferase (SGPT/ALT)	21	12-78	units/L
Alkaline phosphatase (ALKP) total	89	50-390	units/L

**Table 2:** Complete blood count showing normal results

Test	Result	Reference	Units
White blood cell (WBC)	14.5	6.2-19.9	K/mm^3^
Red blood cell (RBC)	4.9	4.5-6.30	mil/mm
Hemoglobin (Hb)	14.7	12.1-15.9	G/DL
Hematocrit (Hct)	41.9	35.8-46.9	%
Mean corpuscular volume (MCV)	86	80-100	um^3^
Mean corpuscular hemoglobin (MCH)	30	27-3	pg
Mean corpuscular hemoglobin concentration (MCHC)	35	32-36	%
Red cell distribution width (RDW)	12	11.5-14.5	%
Platelet count	292	140-450	K/mm^3^
Granulocytes, %	80.1	54-80.7	%
Absolute neutrophils, n	11.6		K/mm^3^
Absolute lymphocytes, %	12.4	9.6-32.6	%
Absolute monocytes, %	7	0-8	%
Absolute eosinophils, %	0.1 (L)	0.3-5.4	%
Absolute basophils, %	0.1	0.1-2.6	%
Immature granulocytes, %	0.3		%

**Table 3:** Lab results showing elevated D-dimer levels

Test	Result	Reference	Units
D-dimer quantitative	3.24 (H)	0.19-0.59	mg/L
Procalcitonin	<0.05	0.0-0.25	ug/L
Lactic acid	2	0.4-2.0	mmol/L
Brain natriuretic peptide (BNP)	<5	0-100	pg/mL
Human chorionic gonadotropin (HCG) serum quantitative	<1	0-3	mIU/mL
Troponin I	<0.02	0.015-0.044	ng/mL

**Table 4:** Urinalysis showing normal results

Test	Result	Reference
UA color	Straw	Yellow
UA appearance	Hazy	Clear
UA glucose	Negative	Negative
UA bilirubin	Negative	Negative
UA ketones	Negative	Negative
UA specific gravity	1.011	1.010-1.030
UA blood	6	5.0-8.0
UA pH	Negative	Negative
UA protein	Negative	
UA urobilinogen	Negative	Negative
UA leukocyte esterase	Negative	Negative
UA microscopic	NO (A)	

**Table 5:** Urine toxicology screen showing normal results

Test	Results	Reference
UR cocaine	Negative	Negative
UR tetrahydrocannabinol (THC)	Negative	Negative
UR amphetamine	Negative	Negative
UR tricyclic antidepressants (TCA)	Negative	Negative
UR barbiturate	Negative	Negative
UR benzodiazepines QL	Negative	Negative
UR methadone QL	Negative	Negative
UR opiates QL	Negative	Negative

After completing an initial history and physical examination in the ED, differential diagnosis included the following:

● Acute pulmonary embolism due to recent start of oral contraceptive pills.

● Syncope with a vasovagal or cardiac origin.

● Seizure.

Furthermore, having an elevated D-dimer, as well as the patient’s dyspnea, chest pressure, cough, and tachycardia made acute pulmonary embolism top of the differential diagnosis. However, CT angiography of the chest was negative. Likewise, with a combination of bystander description, laboratory workup being non-contributory, and CT of the brain within normal limits, clinical suspicion of a seizure was decreased. Lastly, cardiogenic cause of syncope was unlikely based on EKG interpretation and laboratory results. The patient remained in stable condition, with no indication for hospital admission, and was discharged from the ED with an impression of syncope due to a vasovagal reaction, which may have been triggered by a combination of dehydration, skipping a meal, and a bout of coughing during softball practice.

Her mother contacted a pediatrician and scheduled an appointment for further evaluation the next day. Six hours prior to the office visit the following day, the patient mentioned feeling faint while walking down the stairs at home, with no loss of consciousness, which quickly resolved without any interventions. The patient’s mother also complained that the patient had a low-grade fever two days prior to the initial visit to the ED and denied having a history of asthma. On review of systems, she continued to endorse a persistent, non-productive cough, chest pressure, and shortness of breath, and had developed a sore throat. All other systems were non-contributory. Vital signs, in the supine position, were as follows: temperature of 98.0o F, pulse rate of 82 beats per minute, respiratory rate of 16 breaths per minute, and blood pressure of 116/72 mmHg. S1/S2 heart sounds were present but without murmurs. Pulmonary examination demonstrated clear breath sounds bilaterally. Five minutes later in the sitting position, pulse rate was 84 beats per minute and blood pressure was 112/70 mmHg. The patient was started on empiric antibiotic azithromycin for the treatment of suspected atypical community-acquired pneumonia (CAP) and was also prescribed an albuterol inhaler for suspected bronchospasm caused by cough syncope. At that time, she had blood taken to test for TSH, T4, and Mycoplasma pneumoniae IgM Ab.

On a follow-up visit three days later, test results showed Mycoplasma pneumoniae IgM Ab level of 1,947 U/mL (reference: 0-769 U/mL), and TSH and T4 within normal limits (Table 6). All of her symptoms, including persistent, non-productive cough, chest pressure, shortness of breath, and throat soreness, had resolved. She was told to finish her course of azithromycin and use an albuterol inhaler as needed before softball practice.

**Table 6:** Lab results showing elevated Mycoplasma pneumoniae IgM Ab

Test	Result	Reference	Units
Thyroid-stimulating hormone (TSH)	1.860	0.450-4.50	uIU/mL
Thyroxine (T4)	8.8	4.5-12.0	ug/dL
Mycoplasma pneumoniae IgM Ab	1947 (H)	0-769	U/mL

## Discussion

CAP continues to be a significant cause of morbidity across the world. Even with improvements in early detection and disease management, studies show that the economic burden of CAP in adults in the United States is upwards of $17 billion annually [1]. The number is even higher if you include the pediatric populations. Furthermore, atypical CAP usually presents with less severe symptoms and often goes untreated. This requires physicians to have a broad knowledge base and often a high index of suspicion in order to recognize various signs and symptoms of atypical pneumonia.

At the pediatrician's office, the patient primarily endorsed a history of low-grade fever, persistent and non-productive cough, chest pressure, shortness of breath, and sore throat. This steered the clinical suspicion toward the respiratory system. Atypical CAP was high on the differential despite a normal chest X-ray in the ED (Figures 1, 2). This is because at an early stage of pneumonia, chest X-ray can often be normal and not show the typical presentation of atypical pneumonia, that is, diffuse reticulonodular perihilar infiltrates [2-3]. This requires keen judgment of the physician to often diagnose atypical pneumonia clinically.

In the patient’s age group, the most common agent for atypical CAP is Mycoplasma pneumoniae. Primary clinical manifestations of Mycoplasma pneumoniae include upper respiratory tract infection and pneumonia. Other known non-respiratory manifestations are mucocutaneous disease (mild erythematous maculopapular rash, urticaria, etc.) and cold agglutinin hemolysis [4-5].

The pathophysiology behind Mycoplasma pneumoniae causing an elevated D-dimer level is not fully understood. Current theories postulate that the disruption of the vascular endothelial membrane and imbalance between coagulation factors caused by direct invasion by the organism, toxin production, immune-mediated response, or cytokine production can tip the scale toward a hypercoagulable state [6]. Even though unnecessary for diagnostic purposes, a coagulation profile would have also been beneficial to help explain the reason behind the elevated D-dimer level in our patient in the ED.

Macrolides, in particular azithromycin, are considered to be the treatment of choice for atypical pneumonia, especially with Mycoplasma pneumonia being the causative agent. Tetracyclines, such as doxycycline, and respiratory fluoroquinolones, such as moxifloxacin or levofloxacin, can also be used as alternative treatments, especially in areas with high macrolide resistance [7]. Our patient showed significant improvement even after two days of azithromycin 250 mg orally once daily but was advised to finish the full five-day course. She was also prescribed an albuterol inhaler 2.5 mg (two puffs) inhaled orally twice a day and 20-30 minutes before each softball practice.

## Conclusions

Atypical CAP presenting with syncope is often difficult to diagnose, especially when syncope is not a typical symptom of atypical CAP. It is vital to increase the index of suspicion among clinicians of this unusual presentation of atypical pneumonia in adolescents and emphasizes the value of obtaining a thorough history from the patient and family and performing a detailed physical examination.
